# In vitro glycemic index, acrylamide content, and some physicochemical and sensorial properties of special dried bread (Peksimet) enriched with einkorn wheat (*Tiriticum monococcum* L.) flour

**DOI:** 10.1007/s13197-024-06035-8

**Published:** 2024-08-18

**Authors:** Ferhat Yuksel, Sümeyye Çağlar

**Affiliations:** 1 Food Engineering Department, Faculty of Engineering and Natural Science, Gumushane University, 29100 Gumushane, Turkey; 2 Nutrition and Dietetics Department, Faculty of Health Science, Nigde Omer Halisdemir University, 51700 Nigde, Turkey

**Keywords:** Dried bread (Peksimet), Einkorn wheat flour, Functional food, Sourdough bread

## Abstract

In the present study, einkorn wheat flour (*Tiriticum monococcum* L.) was incorporated into a special dried bread (peksimet) formulation produced from sourdough breads at different concentrations (0–10–20–30–40 and 50 g 100 g^−1^) and some physicochemical and nutritional (total dietary fiber, resistant starch, glycemic index, acrylamide content) characteristics and sensory properties of the samples were investigated. The total dietary fiber content of the bread samples ranged from 3.00 to 6.17 g 100 g^−1^. The highest acrylamide content (247.54 µg/kg) was obtained using an einkorn flour level of 40 g 100 g^−1^. Einkorn wheat flour resulted in a significant decrease (from 94.61 to 89.23) in the glycemic index level of the bread samples (*p* < 0.05). Bread enriched with einkorn wheat flour (50.0 g 100 g^−1^) received the highest overall acceptability score. In conclusion, einkorn wheat flour could be used in a special dried bread formulation to enhance its nutritional quality.

## Introduction

Bread is the most consumed basic nutrition source in the world. Bread is an important nutrient product in terms of being cheap, satisfying, and a good source of energy. In general, a bread comprises 50.5% carbohydrates, 37%, 8.7% protein, 3.2% fat, and 2.0% ash, and 100 g of bread provides an average of 270 kcal of energy (Yuksel and Kayacier 2022).

Since ancient times, dough has been prepared by adding flour to products containing lactic acid bacteria, such as yogurt and cheese. Afterwards, new yeast is obtained by leaving this dough to ferment in a warm environment. Then, a new fermentation process is performed with this yeast to make new dough. This process is called sourdough leavening, and the bread obtained by this method is called sourdough bread (Hansen and Schieberle 2005). Bread prepared through the sourdough method has a short shelf life due to the high humidity level it contains. For this reason, these breads are dried and turned into peksimet to increase their shelf life (Axel et al. 2017; Çağlar 2020). Peksimet is a food product that is produced by removing moisture from bread and can remain unspoiled for a long time. The definition of peksimet by the Turkish Language Institution is as follows; “*It is a long-lasting bread that is cut into slices after baking and dried with heat*.” (Çağlar 2020).

One of the most important concerns of the food industry is the shelf life of foods. Bread has a short shelf life because of problems such as staleness and mold (Axel et al. 2017; Yuksel and Kayacier 2022). However, peksimet can be easily consumed as it can be stored for a long time. In addition, peksimet gains new nutritional properties such as high enzyme-resistant starch and low glycemic index during the drying period. (Çağlar 2020).

Siyez (*Triticum monococcum L. subsp. Monococcum)* belongs to the genus Triticum breed. It is referred to as einkorn in the literature. The genetic structure of einkorn, which is accepted as the ancestor of wheat, is a diploid (14 chromosomes). The contents of einkorn wheat are as follows: 11.6% moisture, 65% carbohydrates, 2.8%–4.2% fat, 2.3%–2.8% ash, and 11.83–25.2% protein. In addition, einkorn wheats have high carotenoid (maximum lutein: 8.1 mg/kg), tocopherol (tocol: 77.96 mg/kg), and phenolic contents. Thanks to these compounds, which are known as functional compounds, einkorn products are healthier than (Hidalgo and Brandolini 2014; Loje et al. 2003).

The aim of this study was to enrich peksimet with einkorn flour in order to present a new healthy peksimet product with long-term self-life for the food industry. For this purpose, after drying the breads prepared with sourdough, peksimet samples were collected and the physicochemical, color, bioactive, and sensory properties of these samples were investigated.

## Materials and methods

### Materials and preparation of peksimet

Wheat flour (WF) (12.9 g 100 g^−1^ moisture, 11.1 g 100 g^−1^ of protein, 2.5 g 100 g^−1^ of dietary fiber, 2.9 g 100 g^−1^ of oil, and 1.8 g 100 g^−1^ of ash), einkorn wheat flour (EWF) (9.95 g 100 g^−1^ of protein, 9.72 g 100 g^−1^ of dietary fiber, 1.75 g 100 g^−1^ of oil, and 64.85 g 100 g^−1^ of carbohydrate) and salt were obtained from a local market (Trabzon, Turkey).

#### Sourdough

First, 75 g WF and 75 g EWF were mixed with 150 mL water, and the dough was prepared with a mixer for 10 min (Kitchen Aid Professional 600, MI, USA). The dough was then covered with a cloth to rest for 24 h. Second, half of the dough was mixed again with 75 g of WF, 75 g of EWF, and 150 mL water, and the dough was then prepared with a mixer for 10 min (Kitchen Aid Professional 600, MI, USA). Afterwards, the dough was rested to cover with a wet cloth for 24 h. The second process was continued for 10 days, and then the sourdough was prepared. The prepared sourdough was stored at 4 °C and then used for peksimet production (Caglar 2020).

#### Sourdough breads

The experimental formulation of peksimet is presented in Table 1. According to the experimental plan, mixtures containing WF and EWF in various ratios were prepared, and the mixtures were homogenized for 5 min. Also, the 20 ± 2 g sourdough, 1.0 ± 0.2 g salt and 70 ± 5 mL water were added at the same ratios to all dough formulations. After the addition of water, the dough was kneaded for 10 min. The obtained dough was kneaded every 30 min for 2 ± 0.5 h with a wet cloth cover. After, the doughs were shaped as rounds and then the doughs were put on the trays with the surface of doughs covered with flour and then the doughs were rested for 16 h at + 4 °C refrigerator. The doughs were then taken from the refrigerator and rested for 1 h at room temperature. After that, the prepared doughs were baked for 45 min at 180 °C. Finally, the breads were rested at room temperature for 30 min and then the sourdough breads were prepared (Caglar 2020).

**Table 1:** Formulation of the peksimet samples

Samples	WF (g 100 g^−1^)	EWF (g 100 g^−1^)	Sourdough (g 100 g^−1^)	Salt (g 100 g^−1^)	Water (mL)
Control	100	0	20 ± 2	1.0 ± 0.2	70 ± 5
P1	90	10			
P2	80	20			
P3	70	30			
P4	60	40			
P5	50	50			

#### Peksimet

The rested and cooled breads were sliced into 103.5 cm pieces and then dried for 24 h at 50 °C in the oven. After that, some of the dried breads were separated for sensory analyses, and the other dried breads were milled as flour with a blender (Mmmrp 1000, Bosch, Germany) for all analyses. The milled Peksimet flour was stored in at + 4 °C refrigerator during all analyses (Caglar 2020).

### Some physicochemical properties

The dry matter, ash, and oil content of the peksimet samples were  determined according to official procedures (AOAC 2000) by the oven drying method using an oven, the dry burning method using a furnace, and the Soxhlet extractor, respectively. A water activity measurer (Decagon, USA) was used to determine the water activity level of peksimet samples (Durmaz and Yuksel 2021). The protein content of the peksimet samples was determined by the Kjeldahl method (AOAC 2000).

The acrylamide content of the peksimet samples was obtained according to the procedure described by Gokmen et al. (2005).

### Color properties of the peksimet sample

Color (L*, a*, and b*) of peksimet samples was measured using a colorimeter (Lovibond, England) (Yuksel 2019).

### Total dietary fiber, resistant starch, non-resistant starch, and total starch and glycemic analysis

Total dietary fiber (TDF) was determined using the assay method for Megazyme TDF (K-TDFR-100A, Ireland). Resistant starch (RS) content of the peksimet samples was determined according to the method described by Goni et al. (1997). In addition, non-resistant starch (NRS) and total starch (TS) content (in vitro) of the peksimet samples were determined using the Megazyme analytical procedure.

The method described by Goni et al. (1997) was used to determine the GI content of the samples. The total starch hydrolysis (TSH) content of the peksimet sample was calculated using the following formula (Eq. 1):

1 $$\begin{aligned} {\text{TSH }}\left( \% \right) & = \left[ {{{\left( {{\text{released glucose weight}} \times {16}0/{182}} \right)} \mathord{\left/ {\vphantom {{\left( {{\text{released glucose weight}} \times {16}0/{182}} \right)} {\left( {\text{total starch weight in bread sample}} \right)}}} \right. \kern-0pt} {\left( {\text{total starch weight in bread sample}} \right)}}} \right] \\ & \quad \times 100 \\ \end{aligned}$$

A nonlinear model for the kinetic analysis of starch digestion in vitro was used according to the method described by Goni et al. (1997). The GI of the samples was calculated using the following equation (Eq. 2):

2 $${\text{GI}} = {39}.{71} + 0.{549}*{\text{HI}}$$

### Sensory properties

The sensory properties of peksimet samples were determined by a panel comprising 25 members (graduate students and academic staff of the Food Engineering Department at Gumushane University, Gumushane, Turkey) using a 9-point scale (1: undesired and 9: desired). The taste, odor, color, firmness, and overall acceptability of the peksimet samples were determined. Drinking water was used to clean the panel members’ palates before proceeding to the next sample (Durmaz and Yuksel 2021).

### Statistical analysis

Statistical data of the peksimet sample were expressed using the general linear model procedure with SAS version 8.2 software packages (SAS 2002, SAS Institute Inc., Cary, NC, USA). Means were divided by ANOVA analysis, and statistical significance was denoted at the 0.05 p value.

## Results and discussion

### Some physicochemical properties of peksimet samples

The some physicochemical properties (dry matter, ash, oil, water activity and protein) of the peksimet samples are given in Table 2. The dry matter content of the peksimet samples ranged from 91.28 to 90.99 g 100 g^−1^ and maximum and minimum dry matter contents were obtained in the control and P2 samples (containing 80 g 100 g^−1^ WF and 20 g 100 g^−1^ EWF), respectively. The dry matter contents of the final peksimet samples were significantly impacted by EWF (*P* < 0.05, Table 2). There was 0.82 g 100 g^−1^ difference between the maximum and minimum dry matter content of the peksimet sample, and the main cause for the difference can be explained with the protein and dietary fiber content of EWF. The protein and dietary fiber contents of EWF were 9.95 g 100 g^−1^ and 9.72 g 100 g^−1^, respectively. As can be seen, the protein and dietary fiber content of EWF are very high and could affect the moisture content of samples because the water holding capacities of EWF were high. Similar results were reported for bread enriched with cumin and caraway seeds and By-Product flour (Ahmad et al. 2015). Emeksizoğlu, (2016) reported that the dry matter content of noodle and flatbread produced from einkorn flour were 86.45–89.19% and these results were found to be similar to the dry matter content of peksimet samples in the present study.

**Table 2:** Some physicochemical properties of the peksimet samples

Samples	Dry matter (g 100 g^−1^)	Ash (g 100 g^−1^)	Oil (g 100 g^−1^)	Water activity (a_w_)	Protein (g 100 g^−1^)
Control	91.81 ± 0.15^a^	1.15 ± 0.11^d^	5.79 ± 0.71^a^	0.38 ± 0.00^d^	9.29 ± 0.07^c^
P1	91.66 ± 0.04^ab^	1.25 ± 0.01^ cd^	3.31 ± 0.27^b^	0.38 ± 0.00^d^	9.54 ± 0.13^bc^
P2	90.99 ± 0.02^b^	1.31 ± 0.02b^cd^	2.32 ± 0.45^b^	0.47 ± 0.00^b^	9.48 ± 0.06^c^
P3	91.26 ± 0.04^ab^	1.43 ± 0.01^ab^	2.61 ± 0.08^b^	0.46 ± 0.00^c^	9.79 ± 0.02^ab^
P4	91.24 ± 0.09^ab^	1.37 ± 0.07^abc^	1.18 ± 0.10^c^	0.48 ± 0.00^a^	10.02 ± 0.08^a^
P5	91.28 ± 0.03^ab^	1.52 ± 0.10^a^	1.16 ± 0.44^c^	0.48 ± 0.00^a^	9.88 ± 0.20^a^

^a–d^Values within a column with different superscript letters are different (*P* < 0.05)

Meaningful increases in the ash amount of the peksimet samples were described when the amount of EWF was increased in the formulation (*P* < 0.05). The highest and lowest ash contents of the peksimet samples were determined as 1.15 and 1.52 g 100 g^−1^, respectively (Table 2). When 50% of EWF was added to the recipe, the ash level of the peksimet samples increased by 38%. The total dietary fiber content of EWF was found to be 9.72 g 100 g^−1^. In the literature, the ash content of EWF was found to be 2.4% (Loje et al. 2003), 2.0% (Yuksel and Campanella 2018) 2.34% (Brandolini et al. 2008), and 2.48% (Emeksizoğlu 2016). Therefore, it could be speculated that the main reason for the increase in the ash level in the peksimet samples with increasing EWF content could be related to the fact that EWF already contains high content of dietary fiber and ash. Yuksel and Campanella (2018) reported that the ash content of dough-enriched einkorn, cranberry, and potato flour were 0.8–1.7 g 100 g^−1^ and these results were similar to the ash content of peksimet samples in the present study. In another similar study, they found the ash content of bread enriched with einkorn flour to be 1.09–1.57 g 100 g^−1^ (Cankurtaran-Komürcü and Bilgiçli 2023).

The maximum and minimum oil contents of the peksimet samples were 5.79 and 1.16 g 100 g^−1^, respectively (Table 2). The highest oil amount was determined in P1 (including 0 g 100 g^−1^ EWF), whereas the lowest oil content was found in P5 (including 50 g 100 g^−1^ EWF). Significant reductions in the amount of oil in the peksimet samples were determined (*P* < 0.05). The oil contents of WF and EWF were determined to be 3.00 and 1.75 g 100 g^−1^, respectively. In the literature, the oil contents of EWF were found to be 2.48% (Abdel-Aal et al. 1995) and 2.19% (Emeksizoğlu 2016). The main reason for the decrement in the oil content of peksimet samples with increasing EWF content is that the EWF is low in oil amount. Hidalgo et al. (2020) reported that the oil content of pasta produced from einkorn flour was 1.87 g 100 g^−1^ and these results were explained to be similar to the oil content of peksimet samples containing 50 g 100 g^−1^ EWF in the present study. In another similar study, they found the oil content of bread enriched with einkorn flour to be 1.28–1.50 g 100 g^−1^ (Cankurtaran-Komürcü and Bilgiçli 2023).

When EWF was added to the formulation, significant increases in the water activity (a_w_) of the peksimet samples were also observed (*P* < 0.05). As shown in Table 2, the a_w_ contents of the peksimet samples varied from 0.38 to 0.48 a_w_. The maximum a_w_ content of the peksimet samples was determined in the P5 sample (containing 50 g/100 g EWF). Even though there were increments in the a_w_ of peksimet samples, these increments were very small (by 0.10 a_w_), and all water activities of samples were under 0.60 a_w_. Generally, if the water activity of a food product is under 0.60, the food product could be safely consumed because it was not virtually exposed to any microbial contamination (Borremans et al. 2020). Thus, the peksimet- enriched EWF could be safely consumed. Jakubczyk et al. (2008) reported that the water activities of crisp breads were 0.030–0.255 a_w_ for rye bread and 0.039–0.319 a_w_ for fiber bread, and these results were similar to the water activity of peksimet samples in the present study.

Meaningful increases were also found in the protein levels of the peksimet samples when EWF was included in the formulation (*P* < 0.05). The lowest (9.29 g 100 g^−1^) protein content was observed in the control samples, whereas the highest protein content (10.02 g 100 g^−1^) was found in the P4 sample containing 40 g 100 g^−1^ EWF. The protein content of EWF was determined to be 9.95 g 100 g^−1^. In the literature, the protein contents of EWF were found to be 14.6% (Abdel-Aal et al. 1995) and 13.8% (Loje et al. 2003). The protein content of EFW used in this study was lower than that reported in the literature because the bran of einkorn wheat was separated from the flour. Nevertheless, the protein content of peksimet samples slightly increased by adding EWF without bran. Keçeli et al. (2021) reported that the protein contents of einkorn wholemeal flour and bread wheat flour were 16.3% and 12.3%, respectively, and these results demonstrated that the protein content of the einkorn wholemeal flour was higher than that of the bread wheat flour due to the protein content of the bran of einkorn. In addition, they reported that the protein contents of samples increased with the addition of einkorn flour into the formulations. Nakov et al. (2018) observed that the addition of einkorn flour increased the protein content of cookies. Aktaş et al. (2008) reported that the protein content of peksimet was 12.10%, and these results were similar to the protein content of peksimet samples in the present study.

### Instrumental color properties of the peksimet samples

The mean values of the instrumental color properties of the peksimet samples are shown in Table 3. The EWF content had a significant effect on the color properties of the peksimet samples (Table 3). The L* value ranged from 77.81 to 64.20, and the L* content of the peksimet samples decreased with the addition of EWF into formulation (*p* < 0.05). The highest L* value was obtained without EWF (control sample), whereas the lowest L* value was obtained in the P4 sample containing 40 g 100 g^−1^ EWF. The a* value varied from 1.79 to 5.03, and the a* content of the peksimet samples increased with the addition of EWF into formulation (*p* < 0.05). The minimum a* value was obtained without EWF (control sample), whereas the maximum a* value was obtained in the P4 sample containing 40 g 100 g^−1^ EWF. The b* value varied from 22.33 to 25.80, and the b* content of the peksimet samples increased with the addition of EWF into formulation (*p* < 0.05). The lowest b* value was obtained without EWF (control sample), whereas the highest b* value was obtained in the P5 sample containing 50 g 100 g^−1^ EWF.

**Table 3:** Color properties of the peksimet samples

Samples	L*	a*	b*
Control	77.81 ± 0.52^a^	1.79 ± 0.27^d^	22.33 ± 0.28^c^
P1	74.42 ± 0.41^b^	3.83 ± 0.32^c^	22.36 ± 0.46^c^
P2	72.67 ± 1.36^c^	3.77 ± 0.48^c^	24.22 ± 1.00^b^
P3	69.54 ± 1.24^d^	4.37 ± 0.56^b^	24.17 ± 0.53^b^
P4	64.20 ± 0.89^f^	5.03 ± 0.41^a^	24.57 ± 0.92^b^
P5	66.78 ± 2.26^e^	5.01 ± 0.53^a^	25.80 ± 0.75^a^

^a–f^Values within a column with different superscript letters are different (*P* < 0.05)

The EWF negatively impacted the L* values while positively impacted the a* and b* values of the peksimet samples. Yuksel and Campanella (2018) reported that the color properties (L*, a* and b*) of wheat flour and einkorn flour were 91.6–0.8–12.3 and 78.6–3.3–15.4, respectively. For this reason, it could be speculated that the main cause of a decrease in the L* content and increment in the a* and b* contents in the peksimet samples with increasing levels of EWF could be related to the fact that EWF contains high amounts of color pigments such as carotenoids. Similar findings were reported for bread and cookies enriched with einkorn flour (Keçeli et al. 2021; Nakov et al. 2018). Hidalgo et al. (2006) reported that the yellow pigment content of einkorn flour was derived from carotenoids (lutein), and the lutein content of einkorn flour was observed to be 8.1 mg/kg. In addition, the drying temperature positively impacted the redness and yellowness of the peksimet samples enriched with EWF. This finding may be related to the Caramelization and Maillard reactions.

### Nutritional properties of peksimet samples

Meaningful increases were found in the TDF contents of the peksimet samples when EWF was included in the formulation (*P* < 0.05). The lowest TDF amount (3.00 g 100 g^−1^) was observed in the P1 samples, whereas the highest TDF (6.17 g 100 g^−1^) was determined in the P5 sample containing 50 g 100 g^−1^ EWF (Table 4). The TDF content of EWF was determined as 9.72 g 100 g^−1^. In the literature, the TDF contents of EWF were found to be 8.7 g 100 g^−1^ (Loje et al. 2003) and mean 8.7 g 100 g^−1^ (Hidalgo and Brandolini 2014). Therefore, it could be speculated that the main cause of an increase in the TDF content in the peksimet samples with increasing amount of EWF could be related to the fact that EWF already contains a high amount of dietary fiber. Similar results were reported by Cankurtaran-Kömürcü and Bilgiçli (2023), who determined the total dietary fiber content of bread to be 3.07–4.58 g g100^−1^.

**Table 4:** Nutritional properties of the peksimet samples

Samples	TDF (g 100 g^−1^)	RS (g 100 g^−1^)	NRS (g 100 g^−1^)	TS (g 100 g^−1^)	Acrylamide (mg/kg)	GI	HI
Control	4.39 ± 0.01^b^	2.10 ± 0.40^a^	76.81 ± 0.99^a^	78.92 ± 0.59^a^	180.26 ± 9.24^c^	94.61 ± 0.01^a^	99.95 ± 0.01^a^
P1	3.00 ± 0.27^c^	1.67 ± 0.08^ab^	80.64 ± 01.00^a^	82.32 ± 0.92^a^	195.43 ± 12.21^bc^	89.77 ± 0.29^bc^	91.18 ± 0.53^bc^
P2	3.60 ± 0.57^bc^	1.54 ± 0.09^b^	80.82 ± 1.35^a^	82.36 ± 1.25^a^	239.32 ± 12.73^ab^	90.72 ± 0.09^b^	92.91 ± 0.19^b^
P3	3.80 ± 0.27^bc^	1.54 ± 0.01^b^	81.71 ± 3.51^a^	83.25 ± 3.50^a^	218.60 ± 2.74^a^	90.70 ± 0.48^b^	92.88 ± 0.87^b^
P4	5.80 ± 0.27^a^	1.57 ± 0.00^b^	80.26 ± 1.91^a^	81.83 ± 1.91^a^	247.54 ± 16.10^a^	88.47 ± 0.02^d^	88.81 ± 0.05^d^
P5	6.17 ± 0.28^a^	1.56 ± 0.26^b^	80.25 ± 4.07^a^	81.82 ± 3.81^a^	236.41 ± 11.34^a^	89.23 ± 0.77^ cd^	90.22 ± 1.40^ cd^

*TDF* Total dietary fiber, *RS* Resistant starch, *NRS* Non-resistant starch, *TS* Total starch, *GI* Glycemic index and *HI* Hydrolysis index

^a–d^Values within a column with different superscript letters are different (*P* < 0.05)

The RS, NRS, and TS values of the peksimet samples are shown in Table 4. The RS content of the samples ranged between 1.54 (P2 sample) g 100 g^−1^ and 2.10 (control sample) g 100 g^−1^ a significant statistical difference between control samples (*p* < 0.05) and other samples, whereas no significant statistical difference from P1 to P5 samples (*p* > 0.05). RS in bread occurs with hydrogen bound between amylose and amylopectin of starch after retrogradation. However, the RS could be decreased with an increase in the protein and oil content of the bread samples because hydrogen binding does not occur. The protein and oil could work as a barrier material for the hydrogen bound between amylose and amylopectin for the formation of RS (Haralampu 2000). Therefore, it could be speculated that the main cause of a decrement in the RS content in the peksimet samples with enhancing content of EWF could be related to the fact that einkorn wheat flour already includes a high protein content. In addition, some studies have demonstrated that the RS content of einkorn is lower than that of wheat bread (Brandolini et al. 2011; Hidalgo and Brandolini 2014). The NRS content of the samples ranged between 76.81 (control sample) g 100 g^−1^ and 81.71 (P3 sample) g 100 g^−1^ with no significant statistical difference (*p* > 0.05). The highest TS value (83.25 g 100 g^−1^) was obtained in the P3 sample including 30 g 100 g^−1^ EWF while the lowest TS (78.92 g 100 g^−1^) value was obtained in the control sample containing 0 g 100 g^−1^ EWF. The TS of the samples was not significantly affected by EWF fortification (*p* > 0.05). In the literature, the TS contents of EWF were found to be mean 62.2 g 100 g^−1^ (Kulathunga et al. 2021) and 65.5 g 100 g^−1^ (Hidalgo and Brandolini 2014). Therefore, the TS content of the peksimet samples was affected by the starch content of WF and EWF.

The acrylamide content of the peksimet samples varied from 180.26 to 247.54 mg/kg (Table 4). A significant increase was found in the acrylamide level of the peksimet samples when EWF was added (*P* < 0.05). In addition, the acrylamide content of the samples was affected by the drying temperature and time (24 h at 50 °C). The lowest acrylamide content was observed in the control samples, whereas the highest acrylamide content was determined in the P4 sample containing 40 g 100 g^−1^ EWF. Acrylamide is an organic compound formed as a consequence of the Maillard reaction, which is a thermal process contaminant (up to 120 °C) produced via a series of step-by-step reactions (non-enzymatic) between glucose, fructose, and asparagine amino acids (Deribew and Woldegiorgis 2021; Yuksel and Kayacier 2022). The asparagine content of common wheat and einkorn wheat was determined to be 292 mg/kg and 996 mg/kg (Yıltırak et al. 2021). In addition, the glucose and fructose contents of refined wheat and whole einkorn were determined to be 0.69 g/kg and 5.09 g/kg, respectively, and the acrylamide content of whole einkorn was determined to be higher than that of refined wheat (Çelik and Gökmen 2020). As a result, it is possible to hypothesize that the fact that EWF already contains significant amounts of reducing sugars and asparagine amino acids is the primary factor contributing to an increase in the acrylamide content in the samples with increasing amounts of EWF. Crawford et al. (2019) found acrylamide contents between 101 and 504 μg/kg in wheat-based matzo breads baked rapidly at high temperature (400 °C) until a crispy texture was obtained.

**Fig. 1:**
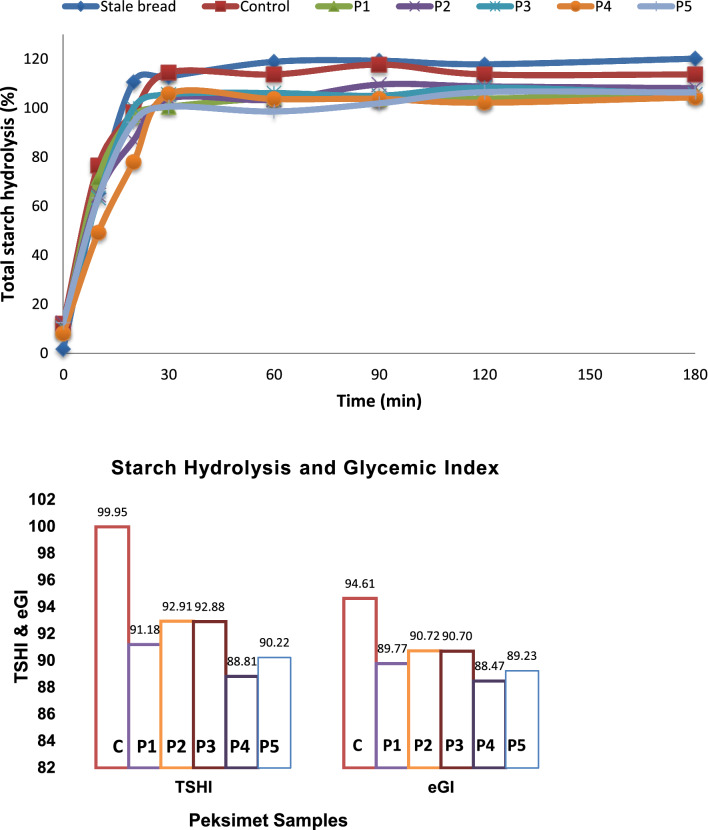
Total starch hydrolysis and glycemic index of the peksimet samples

The maximum GI and HI contents of the peksimet samples were determined as 94.61 and 99.95, respectively, while the minimum GI and HI contents of the peksimet samples were observed as 88.47 and 88.81, respectively (Table 4, Fig. 1). A significant decrease was found in the GI and HI contents of the samples when EWF was included in the formulation (*P* < 0.05). The lowest GI and HI contents were observed in the P4 samples containing 40 g 100 g^−1^ EWF. A similar finding was reported for cookies enriched with einkorn whole meal flour (Nakov et al. 2018). Marangoni and Poli (2008) reported that the GI content of bread and biscuit-enriched dietary fiber was reduced. Another similar study reported by Chang et al. (2014) showed that the GI content of brown rice was lowly determined due to the high fiber content of the brown rice. The GI of foods could be affected by the protein, fiber, and oil content of foods. The high oil content of foods could extend the period of transition of nourishment to the intestine, the high protein content of foods could increase insulin release, and the high protein and fiber content of foods could diminish the digestion of starch (Sayaslan et al. 2016). For this reason, it could be estimated that the main cause of a decrease in the GI and HI content in the samples with increasing levels of EWF could be related to the fact that EWF contains high protein and TDF content. The International Standards Organization classifies glycemic index readings of 70 and above as high, 56–69 as medium, and 55 and below as low. The results show that even though the glycemic index of the bread decreased when einkorn wheat was added, the bread’s total glycemic index value was still high. A study on cookies found no significant difference in digestion speeds between cookies made with 100% wheat flour and 100% einkorn flour (Nakov et al. 2018). Another study found that einkorn wheat had glycemic index values ranging from 83.0 to 85.7, ranking the grain in the high glycemic index class (Kulathunga and Simsek 2023).

### Sensorial properties of peksimet samples

The color, firmness, taste, odor, and overall acceptability of the enriched peksimet were evaluated by the panelists. The mean values of the sensorial color properties of the peksimet samples are shown in Table 5. Significant increases were not found in the color, firmness, taste, and odor contents of the peksimet samples (*P* > 0.05), whereas considerable increases were found in the overall acceptability contents of the samples when EWF was included in the formulation (*P* < 0.05). When the overall liking scores of the sensory analysis of peksimet samples were examined, it was observed that the addition of EWF increased the overall liking. The maximum overall acceptability content of the peksimet sample was determined to be 7.67 in P5, whereas the minimum overall acceptability content of the peksimet sample was 6.73 in the control sample. The sensory analyses revealed that the panelists gave higher sensory scores to the peksimet with 50 g 100 g^−1^ of EWF, compared with those of the peksimet with 0 g 100 g^−1^ of EWF. It can be concluded that the addition of EWF into the peksimet formulation up to 50 g 100 g^−1^ don’t have any negative effects on the sensorial properties of the enriched peksimet. Also, firmness is essential for dried foods. Although the firmness of the peksimet samples was not determined significantly, there was an increment between the control samples and the P5 sample. The maximum firmness content of the peksimet sample was determined as 7.66 in P5, whereas the minimum firmness content of the peksimet sample was observed as 7.00 in the control sample. Similar findings have been reported for toasted rusk rolls (Primo-Martin et al. 2008).

**Table 5:** Sensory properties of the peksimet samples

Samples	Color	Firmness	Taste	Odor	Overall acceptability
Control	7.20 ± 1.53^a^	7.00 ± 1.75^a^	6.93 ± 1.55^a^	7.07 ± 1.62^a^	6.73 ± 1.37^b^
P1	7.13 ± 1.47^a^	7.53 ± 1.00^a^	7.40 ± 2.19^a^	7.40 ± 1.93^a^	7.46 ± 1.66^ab^
P2	6.86 ± 1.88^a^	7.53 ± 1.73^a^	7.13 ± 1.32^a^	7.27 ± 1.74^a^	7.40 ± 1.65^ab^
P3	7.40 ± 1.55^a^	7.20 ± 1.41^a^	7.26 ± 1.51^a^	6.47 ± 1.62^a^	7.46 ± 1.29^ab^
P4	7.26 ± 1.11^a^	7.20 ± 1.64^a^	6.93 ± 1.22^a^	7.33 ± 0.59^a^	7.60 ± 0.82^a^
P5	7.07 ± 1.66^a^	7.66 ± 1.55^a^	7.66 ± 1.30^a^	7.67 ± 1.63^a^	7.67 ± 1.68^a^

^a–b^Values within a column with different superscript letters are different (*P* < 0.05)

## Conclusion

The peksimet was enriched with EWF to develop a healthy food product. The addition of EWF to the peksimet formulation increased the total dietary fiber content and decreased the glycemic index of the resultant product. The most popular product formulation was determined to be a peksimet sample containing 50 g 100 g^−1^ EWF. Finally, it was shown that EWF may be added to peksimet to improve nutritional quality.
